# Effects of Tobacco Smoking in Pregnancy on Offspring Intelligence at the Age of 5

**DOI:** 10.1155/2012/945196

**Published:** 2012-12-20

**Authors:** Hanne-Lise Falgreen Eriksen, Ulrik Schiøler Kesmodel, Theresa Wimberley, Mette Underbjerg, Tina Røndrup Kilburn, Erik Lykke Mortensen

**Affiliations:** ^1^Section of Epidemiology, Department of Public Health, Aarhus University, Bartholins Allé 2 Aarhus C, 8000 Aarhus C, Denmark; ^2^Department of Clinical Medicine, Center of Functionally Integrative Neuroscience, Aarhus University, 8000 Aarhus C, Denmark; ^3^Department of Obstetrics and Gynaecology, Aarhus University Hospital, 8200 Aarhus N, Denmark; ^4^Section of Biostatistics, Department of Public Health, Aarhus University, 8000 Aarhus C, Denmark; ^5^Department of Economics and Business, National Centre for Register-Based Research, Aarhus University, 8000 Aarhus C, Denmark; ^6^Children's Neurocenter Vejlefjord Rehabilitation Center, 7140 Stouby, Denmark; ^7^Center of Child and Adolescent Psychiatry, Central Denmark Region, Section C, 8240 Risskov, Denmark; ^8^Institute of Public Health and Center for Healthy Ageing, University of Copenhagen, 1353 Copenhagen K, Denmark

## Abstract

The aim of the study was to examine the effects of tobacco smoking in pregnancy on children's IQ at the age of 5. A prospective follow-up study was conducted on 1,782 women, and their offspring were sampled from the Danish National Birth Cohort. At 5 years of age, the children were tested with the Wechsler Preschool and Primary Scale of Intelligence-Revised. Parental education, maternal IQ, maternal alcohol consumption in pregnancy, the sex and age of the child, and tester were considered core confounders, but the full model also controlled for prenatal paternal smoking, maternal age and Bodymass Mass Index, parity, family/home environment, postnatal parental smoking, breast feeding, the child's health status, and indicators for hearing and vision impairments. Unadjusted analyses showed a statistically significant decrement of 4 points on full-scale IQ (FSIQ) associated with smoking 10+ cigarettes per day compared to nonsmoking. After adjustment for potential confounders, no significant effects of prenatal exposure to tobacco smoking were found. Considering the indisputable teratogenic effects of tobacco smoking, these findings should be interpreted with caution. Still, the results may indicate that previous studies that failed to control for important confounders, particularly maternal intelligence, may be subject to substantial residual confounding.

## 1. Introduction

 The negative effects on health of active as well as passive exposure to tobacco smoking have long been known. A special case of exposure is that of the developing fetus when a pregnant woman is smoking, exposing the fetus to the adverse effects of the numerous toxins contained in tobacco, such as vasoconstriction and hypoxia [1]. Blood concentrations of cotinine, a nicotine metabolite, in exposed newborns indicate that the fetus is exposed to equal—or even higher—levels of nicotine than the smoking mother [2, 3]. The list of known adverse short- and long-term sequelae associated with prenatal exposure to tobacco smoke includes preterm delivery, pre- and postnatal growth restriction [4, 5], congenital malformations, [6] stillbirth, [7] and increased risk of Sudden Infant Death Syndrome [8, 9]. 

Further, tobacco smoking may act as a neuroteratogen through various mechanisms [10]. Nicotine and its metabolite cotinine may alter the function of several neurotransmitter systems, primarily acetylcholine, serotonin, and catecholamines [3]. Functionally, prenatal exposure to tobacco smoking has been associated with cognitive impairments, particularly in attention and linguistic skills presumably related to compromised auditory processing [11–15]. Studies of the effects on general cognitive abilities or intelligence have provided mixed results. Hence, while some studies reported lower IQ scores in exposed individuals [16–20], others reported significant associations to disappear after adjustment for confounders [21–24], and yet others found no association [25]. The overall conclusion regarding the effects of smoking in pregnancy on offspring intelligence thus remains ambiguous, and it is widely debated whether previously reported associations reflect causal relations or rather methodological shortcomings, such as residual confounding [23, 26].

 The purpose of this study was to examine the effects of prenatal exposure to tobacco smoking on psychometric intelligence (IQ) in a large sample of 5-year-old children while taking into account a wide range of important confounders, including parental education, maternal intelligence, and alcohol consumption in pregnancy, and postnatal smoke exposure. 

## 2. Materials and Methods

### 2.1. Study Sample

 The study was a part of the Lifestyle During Pregnancy Study (LDPS) [27], a prospective follow-up study of the effects of various maternal lifestyle factors in pregnancy, primarily intake of alcohol, on motor and cognitive outcomes at the age of 5 years. The LDPS is based on a subsample from the Danish National Birth Cohort [28], a large cohort study with information on 101,042 women and their children, collected by two prenatal and two postnatal telephone interviews. 

The data collection of the LDPS took place from September 2003 to June 2008 the period during which 3,478 mothers and their children were invited to a followup when the child was from 60 to 64 months of age. Of these, 1,782 (51%) participated in a comprehensive three-hour assessment of the child's cognitive ability, including tests of global and specific functions.

Exclusion criteria were multiple pregnancies, inability to speak Danish, impaired hearing or vision likely to compromise the ability to perform the cognitive tests, and congenital disabilities likely to imply mental retardation (e.g., Down's syndrome, infantile autism).

### 2.2. Exposure Measure

 Data on maternal smoking habits in pregnancy was obtained by the first prenatal interview in the DNBC carried out at a median of 17 gestational weeks (range 7–39). The women were asked about their daily and weekly number of smoked cigarettes, and based on this information the women were categorised in three exposure levels (0, 1–9, and 10+ cigarettes per day). The interview also comprised information on smoked pipes, cheroots, and cigars, but none of the participants reported smoking any of these types.

### 2.3. Outcome Measure

 Intelligence was assessed with the Wechsler Primary and Preschool Scales of Intelligence-Revised (WPPSI-R) [29, 30], which is one of the most widely used, standardised measures of intelligence for children of 3 to 7 years. The full WPPSI-R comprises five verbal and five performance (nonverbal) subtests. To reduce the length of the test session, we used three verbal (Arithmetic, Information, and Vocabulary) and three performance subtests (Block Design, Geometric Design, and Object Assembly). This set of subtests was selected taking into consideration (a) correlation with FSIQ and (b) variety in the composition of the test battery which should make it possible to derive verbal and performance IQ in addition to FSIQ.

Standard procedures [30] were used to prorate Verbal IQ (VIQ), Performance IQ (PIQ), and Full-Scale IQ (FSIQ) from this shortened form of the test.

No Danish WPPSI-R norms were available at the time of the study, and consequently Swedish norms were used to derive scaled scores and IQs [31]. Because Swedish norms were used, the theoretical IQ distribution of a mean of 100 and a standard deviation (SD) of 15 cannot necessarily be expected in this sample, and IQ-scores may not be uncorrelated with age. This, however, will not affect internal comparisons made within the sample with respect to effects of smoke exposure.

Testing took place in one of the four major cities of Denmark (Copenhagen, Odense, Aalborg, and Aarhus). Test procedures were standardised in detail and carried out by 10 trained psychologists. Tester differences were taken into account by the inclusion of a categorical variable for tester in the statistical analyses.

### 2.4. Covariates

 The following information was obtained by the prenatal telephone interview and subsequently coded as shown in parenthesis: maternal alcohol consumption during pregnancy (yes/no), parity (0, 1, 2+), maternal pre-pregnancy BMI (weight in kg/(height in m)^2^), and prenatal paternal smoking (yes/no).

A questionnaire administered at the followup provided information on the following variables: maternal marital status (single at either the prenatal interview or followup/with partner at both times), parental education in years (averaged for both parents if paternal information was available, otherwise maternal only), postnatal parental smoking (one or both parents smoked/both were nonsmokers), an index of the quality of postnatal home environment (dichotomised as normal or suboptimal in the presence of two or more of the following adverse conditions: living with only one biological parent, changes in primary care givers, daycare for more than 8 hours/day before age 3, 14+ days of separation from parents, irregular breakfast, maternal depression, and maternal/paternal alcohol intake above the official recommendations from the Danish National Board of Health at the time of the data collection), an index of the child's health status (dichotomised as normal or suboptimal in the presence of any handicaps, illness, diseases and/or medication with potential influence on test performance), and breast feeding (≤1 month, >1 month). To exclude potential undetected impairments, hearing and vision abilities (impaired/normal) of the child were assessed at the follow-up examination, as was maternal IQ; two verbal subtests (information and vocabulary) from the Wechsler Adult Intelligence Scale [32] (WAIS) were used to assess verbal IQ, and the Raven Standard Progressive Matrices [33] provided nonverbal IQ. The raw scores of each test were standardised based on the results from the full sample and weighted equally in a combined score that was restandardised to an IQ scale with a mean of 100 and an SD of 15.

Maternal age was obtained from the Danish Civil Registration System as was the sex and age of the child. Birth weight (grams) and gestational age (days) were obtained from the Danish Medical Birth Registry. 

### 2.5. Data Analysis

 All statistical analyses were conducted with Stata 11 (StataCorp LP, College Station, TX, USA). 

In the LDPS, the higher alcohol categories were oversampled, and consequently all analyses were weighted by sampling probabilities. All statistical tests were two-sided and declared significant at the 5% level. All estimates are accompanied by 95% confidence intervals. The extent of missing values on individual variables ranged from 2 (0.1%) on hearing to 59 (3.3%) on prenatal paternal smoking, with 36 (2.0%) missing values on maternal BMI and 8 (0.4%) missing values on full-scale IQ. For the remaining variables, the extent of missing values was below 0.8 percent. Missing values were imputed based on a model in which variables were modelled from other variables considered predictive. All conclusions were maintained when complete case analysis was conducted (*N* = 1,702–1,774). We report the results from the imputed analyses. All imputations were implemented with the -ice- add-on command, and the built-in -mi estimate-command of Stata 11.

Associations between smoking exposure categories (0, 1–9, 10+) and the continuous FSIQ, VIQ, and PIQ outcome scores were estimated using multiple linear regression. Parental education, maternal IQ, and maternal alcohol consumption during pregnancy plus the child's age at testing, the child's sex, and tester were considered core confounders included as covariates in a separate model. The final model, in addition, included all potential confounders. Birth weight and gestational age were considered potential mediators of the effects of smoking exposure and not included in these main analyses.

Additionally, we analysed the three IQ outcomes dichotomised, using the sample mean minus one SD for the relevant IQ score (FSIQ, VIQ, or PIQ) as cutoff score for subnormal test performance. Because logistic regressions were used in these analyses, we report odds ratios, with the category of IQ above the cut-off as the reference group.

In supplementary analyses, we analysed raw scores of each individual WPPSI-R subtest with linear regression models adjusting for core and all confounders. Potential interactions with smoking exposure were assessed for sex, parental education, and maternal alcohol consumption in pregnancy.

Pairwise correlations between all core and potential confounders were tested. For all continuous covariates, potential quadratic associations with the IQ outcomes were tested. No significant nonlinear associations were found. 

## 3. Results

Table 1 presents sample characteristics. Women, who smoked during pregnancy tended to be younger, have shorter education and lower IQs than nonsmokers. There were significantly higher proportions of single mothers and suboptimal home conditions in the two smoking categories. Children of smokers had lower birth weights than children of nonsmokers and were less likely to have been breast-fed. It should be noted that these differences were unweighted for the stratified sample and thus not representative for the background population. There were slightly more binge drinkers (66.3% versus 61.8%) and slightly fewer smokers (31.6% versus 35.2%) among participants compared to nonparticipants (data not shown), but otherwise no substantial or significant differences were observed. 

 Pairwise, weighted correlations between all core and potential confounders (complete case) showed significant correlations between maternal IQ and parental education (*r* = 0.47), maternal age and parental education (*r* = 0.21), maternal age and parity (*r* = 0.42), paternal smoking at the time of interview and postnatal parental smoking (*r* = 0.42), parental education and maternal BMI (*r* = −0.24), and single mother and home environment (*r* = 0.64). All other coefficients were lower than 0.2 and most were close to zero. 

### 3.1. WPPSI-R

 The unadjusted analyses showed a statistically significant effect of smoking 10+ cigarettes per day, with a decrement of 3.7 FSIQ points (95% CI: −6.1, −1.2) compared to nonsmokers (Table 2). On the subscales, the crude effect was larger on PIQ (mean diff. = −4.0, 95% CI: −7.1, −0.9) than VIQ (mean diff. = −2.5, 95% CI = −4.7, −0.4). In the model including the core confounders (i.e., maternal IQ, parental education, maternal prenatal alcohol consumption, the sex and age of the child, and tester), the effect estimates were substantially reduced and not statistically significant. This pattern was maintained when additional adjustment was made for the potential confounders.

The logistic regression analyses of the IQ outcomes dichotomised at the sample mean minus one SD showed neither statistically significant nor systematic differences between the two exposure categories and the reference group, except for an increased risk of low VIQ in the 1–9 category compared to the reference group that was marginally significant in the unadjusted analysis (OR = 1.78, 95% CI: 1.03, 3.09) (Table 3). After adjustment, this effect was slightly reduced and not statistically significant (OR = 1.65, 95% CI: 0.85, 3.22). 

In the unadjusted analyses of outcomes on the subscales, smoking 10+ cigarettes per day was associated with marginally significant, lower scores on the information (mean diff. = −0.6, 95% CI = −1.1, 0.0) and the arithmetic subtests (mean diff. = −0.6, 95% CI: −1.2, 0.0) (Table 4). Both exposure categories were associated with significantly poorer performance on the geometric design subtest compared to the reference group, with unadjusted effect estimates of −2.0 (95% CI: −3.8, −0.1) and −2.6 (95% CI: −4.7, −0.5) for the 1–9 and the 10+ categories, respectively. Adjustment for core and all confounders did not change the effect estimates, and the effect of 1–9 cigarettes/day was still marginally significant (mean difference = −2.0, 95% CI: −3.8. −0.1) while the effect of 10+ cigarettes per day approached statistical significance (mean difference = −2.0, 95% CI: −4.3, 0.2). Adjustment for birth weight and gestational age did not alter this result. There were no significant associations between exposure status and the remaining subtests at any level of analysis.

Additional adjustment for gestational weeks of interview did not alter any of these conclusions. The supplementary analyses showed no significant interactions between smoking exposure and sex, parental education, or prenatal alcohol exposure. 

## 4. Discussion

 This study confirmed previous consistent findings that smoking in pregnancy covaries with a range of social and family characteristics, including maternal education [34], socioeconomic status [35], maternal age, and marital status [34, 36, 37], while, in this sample, we did not observe the commonly reported association between smoking and alcohol consumption [38, 39].

We found no evidence of an effect of smoking exposure per se on offspring intelligence after adjustment for confounders. Thus, significant effects of smoking 10+ cigarettes per day in pregnancy on the three IQ scales disappeared when adjustment was made for parental education, maternal IQ, and prenatal maternal alcohol consumption. Adjustment for additional covariates did not change this conclusion. A similar pattern applied to scores on some subtests whereas, in the analyses of dichotomised IQ, no significant differences were found.

The overall results of the present study are thus in line with previous studies in which statistical adjustment of potentially confounding factors eliminated an apparent effect of smoking exposure on IQ [21–24, 26, 40–43]. Lundberg et al. [44] addressed the causal effect of prenatal smoking exposure by comparing the intellectual performance (as measured by a military draft board test) of 14,722 pairs of full siblings, only one of which had been exposed to smoking in utero. There were no differences between exposed and unexposed siblings but an increased risk of low test performance for both if the mother had smoked only during her first pregnancy and no difference compared to nonexposed controls for either sibling if she had smoked only during her second. These results support no effect of smoking per se but rather of maternal and familial characteristics although it should be noted that the study only used a rather crude, dichotomous outcome measure. 

By contrast, other studies have reported negative effects of maternal smoking in pregnancy on child IQ [16–20, 45–47]. A series of followups conducted in the Ottawa Prenatal Prospective Study (OPPS) reported significant effects on Full-Scale and/or Verbal IQ at ages 3–4, 9–12, and 13–16 [20, 45, 48]. At the 13–16 year follow-up, prenatal exposure to 16 mg nicotine/day or more per day was associated with an adjusted decrement of 8 FSIQ points on the Wechsler Intelligence Scale for Children. The sample sizes in the OPPS, however, are generally small, and the 13–16 year follow-up included a total of 145 individuals with only 36 individuals in the 16+ category. 

The divergent results may arise from methodological differences, one of the most important being confounder adjustment. As indicated by the results of the present study, socioeconomic position, often measured by education or income, and maternal IQ seem to be particularly important, and the lack of control for each may produce spurious effects of smoking exposure on outcomes such as IQ [26, 40]. 

Adjustment for education alone has been reported to attenuate the association between smoking and outcomes on IQ by 30%–40% [49]. While most studies did control socioeconomic factors to some extent, only few studies have taken maternal IQ into account. Of six studies controlling maternal IQ [17, 22, 26, 40, 42, 50], two studies reported significant effects of smoking [17, 50]; one of these, however, only at age 10 but not at age 5 and in adolescence, and both studies only measured verbal IQ using the Peabody Picture Vocabulary Test. In the present study, separate adjustments for either parental education or maternal IQ reduced the unadjusted effect estimates by 50–60% and resulted in the association being statistically nonsignificant. Reversely, removing both confounders from the fully adjusted model resulted in statistically significant effects that were augmented by approximately 60%.

The present study was based on a large sample size and controlled for important confounders which not all have been included simultaneously in most previous studies. In addition to the already mentioned confounders, we were able to control for paternal prenatal smoking. This variable both accounts for some of the variance related to unmeasured and potentially confounding paternal and familial factors and provides a measure of maternal passive smoking during pregnancy. Validations of self-reported smoking in pregnancy by measures of cotinine levels suggest that self-reports may lead to misclassification, not because women report their own smoking unreliably, but because of passive smoking [51, 52].

Many previous studies have been characterised by small sample sizes resulting in low statistical power. Still, even when including a large number of observations, the risk may still be present of insufficient statistical power to detect potential effects if these are subtle and if many covariates are included. In this study, the close-to-zero effect estimates and the reasonably narrow confidence intervals support the validity of the findings of no-association and speak against a type II error. The effects of maternal smoking on the geometric design subtest could indicate effects on more specific and sensitive cognitive measures. Although this finding is a natural aim for further investigation, an effect confined to a single subtest is of minor relevance in this context, the focus being on general intelligence as an outcome.

Some limitations of the study should be noted. Well-educated women are likely to be overrepresented in the study population [53], and smoking exposure may therefore be of restricted range with respect to heavy exposure. Although the proportion of smokers in this sample (28%) is higher than the prevalence of smoking among Danish pregnant women reported elsewhere [54]—probably reflecting the oversampling of women with a high alcohol intake—only three women reported smoking more than 20 cigarettes per day at the time of interview. Because of this sampling design, the sample was not representative for the background population. This was accounted for statistically by weighting the analyses by the sampling probabilities. 

Sample selection bias due to sample attrition may be present in studies of this type. A comparison of participants and nonparticipants did not indicate any substantial differences on the available measures. Selection bias on variables on which information on nonparticipants was unavailable, however, cannot be excluded. Another potential source of bias are the exclusion criteria, which arguably may exclude children of less-advantaged families, potentially more vulnerable to the harmful effects of smoking exposure. In this study only a total of 24 children were excluded; 22 who were twins, and two who were diagnosed with Asperger's Syndrome. However, to our knowledge Asperger's Syndrome has not been associated with tobacco smoking. In fact, it may be that the isolated effect of smoking exposure per se is more accurately assessed in a sample with fewer competing risks.

 Further, because smoking, particularly in pregnancy, is associated with social stigmatisation, some degree of underreporting may well be present. The resulting misclassification could potentially bias estimates toward null effects.

The age of the children in the study sample implies specific methodological issues. Test reliability and in particular stability are relatively low in children at age from 4 to 5 [55]. For the WPPSI-R, however, reliability coefficients for the present age group for the IQs are very high (0.90–0.96), yet lower for the individual subtests (0.49–0.80) [31]. 

The fact that controlling maternal education and IQ dilute or deplete the association between maternal smoking and child IQ does not per se preclude causality between exposure and outcome [26], and precautions should be taken in interpreting negative results as evidence against harmful effects of smoking in pregnancy on the cognitive development of the child.

## 5. Conclusions

 The adverse effects of smoking on pregnancy outcomes are indisputable, and animal studies have provided basis for assuming that nicotine and/or other components of tobacco and tobacco smoke may affect human brain development in a harmful manner [56]. This study, however, did not show any significant effects on intelligence at age 5 of maternal smoking in pregnancy when adjusting statistically for a number of important confounders not included in many previous studies.

## Figures and Tables

**Table 1:** Sample characteristics across levels of maternal cigarette smoking in pregnancy.

	Average number of cigarettes per day			Total	*P* ^b^
	0	1–9	10+^a^		
Number of participants	1276	263	243	1,782	
Timing of interview (gestational week)	17.0 (13.0/24.0)	16.0 (12.0/24.0)	17.0 (13.0/24.0)	17.0 (13.0/24.0)	0.608
Median number of cigarettes	0	5	13	0	
Maternal age (years); mean (SD)	31.0 (4.2)	30.2 (4.9)	30.5 (4.8)	30.8 (4.4)	0.019
Parity					
0 (%)	50.6	51.5	51.9	50.9	0.800
1 (%)	32.3	33.7	29.2	32.1	
2+ (%)	17.1	14.8	18.9	17.0	
Maternal Body Mass Index (kg/m^2^)	22.7 (19.6/29.0)	22.5 (19.7/28.4)	22.3 (19.2/28.4)	22.6 (19.6/28.7)	0.810
Single mother (%)	9.7	15.9	22.8	12.4	0.000
Parental education (years)	13.0 (11.0/16.5)	12.5 (10.5/15.5)	12.0 (10.5/14.5)	13.0 (11.0/16.0)	0.000
Suboptimal home environment (%)	14.6	25.0	37.3	19.2	
Maternal IQ; mean (SD)	101.6 (14.8)	96.6 (14.8)	95.2 (14.7)	100.0 (15.0)	0.000
Maternal alcohol consumption in pregnancy (%)	52.8	50.8	51.0	52.3	0.760
Paternal prenatal smoking (%)	23.4	57.1	64.9	33.8	0.000
Parental postnatal smoking (%)	16.4	68.6	76.3	32.3	0.000
Child's sex (male, %)	53.4	48.1	47.7	51.8	0.125
Age at testing (years)	5.2 (5.1/5.3)	5.2 (5.1/5.3)	5.2 (5.1/5.3)	5.2 (5.1/5.3)	0.551
Birth weight (grams)	3649.0 (513.1)	3546.4 (492.9)	3424.1 (508.8)	3603.2 (515.5)	0.000
Gestational age (days)	282.0 (268.0/293.0)	282.0 (267.0/292.0)	280.0 (267.0/294.0)	281.0 (267.0/293.0)	0.229
Breast feeding ≤ 1 month (%)	12.7	15.8	21.1	14.3	0.004
Condition/medicine (%)	2.7	3.4	5.3	3.2	0.104
Impaired hearing (%)	4.6	4.2	7.0	4.8	0.229
Impaired vision (%)	2.4	2.3	7.8	3.1	0.000

Data are presented as medians (10/90 percentiles), unless otherwise specified.

^a^Range 10–25 cigarettes/day.

^b^ *P* values for unweighted data.

**Table 2:** Maternal smoking in pregnancy and WPPSI-R^a^ performance.

Average number of cigarettes/day	Crude		Adjusted for core confounders^b^	Adjusted for potential confounders^c^
	Mean score	Mean difference (95% CI)	Mean difference (95% CI)	Mean difference (95% CI)
Full-scale IQ				
0	106.1	Reference	Reference	Reference
1–9	103.3	−2.8 (−5.5, −0.1)	−1.0 (−3.4, 1.4)	−0.9 (−3.6, 1.8)
10+	102.5	−3.6 (−6.1, −1.2)	−1.0 (−3.3, 1.4)	−0.3 (−3.1, 2.5)
Verbal IQ				
0	105.3	Reference	Reference	Reference
1–9	103.2	−2.1 (−4.4, 0.2)	−0.5 (−2.6, 1.6)	−0.6 (−2.8, 1.7)
10+	102.8	−2.5 (−4.7, −0.4)	−0.1 (−2.2, 2.0)	0.2 (−2.1, 2.8)
Performance IQ				
0	105.6	Reference	Reference	Reference
1–9	102.6	−3.0 (−6.4, 0.5)	−1.4 (−4.5, 1.7)	−1.0 (−4.7, 2.7)
10+	101.5	−4.1 (−7.1, −0.9)	−1.7 (−4.6, 1.2)	−1.0 (−4.6, 2.6)

^a^Wechsler Preschool and Primary Scale of Intelligence-Revised.

^b^Parental education, maternal IQ, prenatal maternal alcohol consumption, the child's sex, age at testing, and tester.

^c^Parental education, maternal IQ, prenatal maternal alcohol consumption, the child's sex, age at testing, and tester, maternal age, parity, maternal marital status, prenatal paternal smoking, postnatal parental smoking, breast feeding, maternal prepregnancy BMI, the child's sex, age at testing, health status, family/home environment.

**Table 3:** Maternal smoking in pregnancy and the risk of low IQ.

Average number of cigarettes/day	Crude	Adjusted for core confounders^a^	Adjusted for potential confounders^b^
	OR (95% CI)	OR (95% CI)	OR (95% CI)
Full-scale IQ			
0	1.00	1.00	1.00
1–9	1.60 (0.94, 2.70)	1.39 (0.78, 2.48)	1.52 (0.78, 2.99)
10+	1.31 (0.74, 2.31)	1.02 (0.56, 1.85)	1.06 (0.55, 2.19)
Verbal IQ			
0	1.00	1.00	1.00
1–9	1.78 (1.03, 3.09)	1.49 (0.81, 2.73)	1.65 (0.85, 3.22)
10+	1.19 (0.64, 2.19)	0.90 (0.48, 1.69)	0.94 (0.50, 1.75)
Performance IQ			
0	1.00	1.00	1.00
1–9	1.19 (0.69, 2.05)	1.08 (0.59, 1.95)	1.04 (0.54, 2.03)
10+	1.65 (0.97, 2.79)	1.41 (0.81, 2.48)	1.31 (0.65, 2.62)

^a^Parental education, maternal IQ, prenatal maternal alcohol consumption, the child's sex, age at testing, and tester.

^b^Parental education, maternal IQ, prenatal maternal alcohol consumption, the child's sex, age at testing, and tester, maternal age, parity, maternal marital status, prenatal paternal smoking, postnatal parental smoking, breast feeding, maternal prepregnancy BMI, the child's sex, age at testing, health status, family/home environment.

**Table 4:** Maternal smoking in pregnancy and WPPSI-R^a^ subtest raw scores.

Avgerage number of cigarettes/day	Unadjusted		Adjusted for core confounders^b^	Adjusted for potential confounders^c^
	Mean score	Mean difference (95% CI)	Mean difference (95% CI)	Mean difference (95% CI)
Information				
0	19.3	Reference	Reference	Reference
1–9	19.0	−0.3 (−0.9, 0.3)	−0.1 (−0.6, 0.5)	−0.1 (−0.7, 0.5)
10+	18.7	−0.6 (−1.1, 0.0)	−0.2 (−0.7, 0.4)	−0.1 (−0.7, 0.5)
Vocabulary				
0	21.1	Reference	Reference	Reference
1–9	20.6	−0.5 (−1.6, 0.6)	0.0 (−1.0, 1.0)	0.0 (−1.1, 1.0)
10+	20.8	−0.3 (−1.3, 0.6)	0.6 (−0.3, 1.5)	0.7 (−0.3, 1.8)
Arithmetic				
0	14.9	Reference	Reference	Reference
1–9	14.3	−0.6 (−1.1, 0.2)	−0.3 (−0.9, 0.2)	−0.3 (−0.9, 0.3)
10+	14.2	−0.7 (−1.2, 0.0)	−0.1 (−0.8, 0.5)	−0.1 (−0.8, 0.6)
Object assembly				
0	23.6	Reference	Reference	Reference
1–9	23.3	−0.3 (−1.2, 0.7)	0.0 (−0.9, 0.8)	0.2 (−0.8, 0.1)
10+	23.1	−0.5 (−1.3, 0.3)	0.1 (−0.8, 0.9)	0.2 (−0.7, 0.1)
Block design				
0	24.3	Reference	Reference	Reference
1–9	23.9	−0.4 (−1.7, 0.8)	−0.2 (−1.4, 1.0)	0.0 (−1.4, 1.4)
10+	23.4	−0.9 (−2.3, 0.5)	−0.2 (−1.5, 1.1)	0.1 (−1.5, 1.5)
Geometric design				
0	37.7	Reference	Reference	Reference
1–9	35.8	−1.9 (−3.8, −0.1)	−1.6 (−3.3, 0.0)	−2.0 (−3.8, −0.1)
10+	35.1	−2.6 (−4.7, −0.5)	−1.9 (−4.0, 0.1)	−2.0 (−4.3, 0.2)

^a^WPPSI-R Wechsler Preschool and Primary Scale of Intelligence-Revised.

^b^Parental education, maternal IQ, maternal prenatal alcohol consumption, the child's sex, age at testing, and tester.

^c^Parental education, maternal IQ, prenatal maternal alcohol consumption, the child's sex, age at testing, and tester, maternal age, parity, maternal marital status, prenatal paternal smoking, postnatal parental smoking, breast feeding, maternal prepregnancy BMI, the child's sex, age at testing, health status, family/home environment.
